# Discordance between lactic acidemia and hemodynamics in patients with advanced heart failure

**DOI:** 10.1002/clc.23584

**Published:** 2021-03-18

**Authors:** Nikhil Narang, Mark Dela Cruz, Teruhiko Imamura, Ben Chung, Ann B. Nguyen, Luise Holzhauser, Bryan A. Smith, Sara Kalantari, Jayant Raikhelkar, Nitasha Sarswat, Gene H. Kim, Valluvan Jeevanandam, Daniel Burkhoff, Gabriel Sayer, Nir Uriel

**Affiliations:** ^1^ Advocate Heart Institute Advocate Christ Medical Center Oak Lawn Illinois USA; ^2^ Division of Cardiology, Department of Medicine University of Chicago Medical Center Chicago Illinois USA; ^3^ Second Department of Medicine University of Toyama Toyama Japan; ^4^ Division of Cardiology Columbia University Irving Medical Center New York New York USA; ^5^ Section of Cardiac Surgery, Department of Surgery University of Chicago Medical Center Chicago Illinois USA; ^6^ Cardiovascular Research Foundation New York New York USA

**Keywords:** biomarkers, cardiogenic shock, heart failure, hemodynamics

## Abstract

**Background:**

Elevated lactic acid (LA) levels carry a poor prognosis in patients with shock. Data are lacking on the significance of LA levels in patients with acute decompensated heart failure (ADHF).

**Hypothesis:**

This study assessed the relationship between LA levels, hemodynamics and clinical outcomes.

**Methods:**

This was a retrospective analysis of registry data of 100 advanced heart failure patients presenting for right heart catheterization (RHC) for concern of ADHF. LA levels (normal ≤2.1 mmol/L) were obtained prior to RHC; no significant changes in therapy were made between LA collection and RHC.

**Results:**

Median age was 58 (47.3, 64.8) years; 57% were receiving inotropes prior to RHC. Median pulmonary capillary wedge pressure (PCWP) and cardiac index (CI) were 28 (21, 35) mmHg and 2.0 (1.7, 2.5) L/min/m^2^, respectively. Eighty patients had normal LA prior to RHC. There was no correlation between LA levels and PCWP (R = 0.09, *p* = .38); 63% of the normal LA group had a PCWP >24 mmHg. There was a moderate inverse correlation between LA and CI (R = − 0.40; *p* < .001); 58% of the normal LA group had a CI <2.2 L/min/m^2^. Thirty‐day survival free of death/hospice, inotrope dependence, progression to heart transplant/left‐ventricular assist device implant was comparable between the normal and elevated LA groups (28% vs. 20%; *p* = .17).

**Conclusion:**

In patients presenting with ADHF, normal LA levels do not exclude the presence of depressed CI (a hemodynamic criteria for cardiogenic shock) and may not offer accurate risk stratification. Invasive hemodynamics should not be delayed based on normal LA levels alone.

## 1 INTRODUCTION

Elevated lactic acid (LA, >2.1 mmol/L) levels have long been used as a reliable marker of end‐organ hypoperfusion and clinical deterioration and serves as a useful clinical prognostication biomarker in patients presenting with sepsis.^1^, ^2^, ^3^ Type A lactic acidemia occurs during anaerobic glycolysis resulting in peripheral tissue hypoxia,^4^ while Type B lactic acidemia occurs in disorders where tissue hypoxia is absent including malignancy, metformin overdose, seizures and *B*_2_‐agonist use. In the cardiac intensive care setting, LA levels are often used as surrogate biomarker to monitor response to inotropes or mechanical circulatory support (MCS) in patients presenting with cardiogenic shock.^5^ LA monitoring is also utilized as a clinical metric (when >4 mmol/L) to upgrade status for heart transplantation when hemodynamics are unavailable in patients with worsening chronic heart failure (HF).^6^

Cardiogenic shock is conventionally defined by sustained hypotension (<90 mmHg) and elevated intracardiac filling pressures manifesting with signs of end‐organ hypoperfusion. Seemingly, it would be plausible to conclude a high prevalence of elevated LA levels in the setting of overt cardiogenic shock. However, cardiogenic shock may present in many phenotypes, including vasodilatory, euvolemic, normotensive, right ventricular, and biventricular variants for which accepted definitions do not apply and treatment strategies differ.^7^, ^8^ In a single‐center study, Adamo et al. showed a low prevalence of elevated LA in advanced HF patients prior to left‐ventricular assist device (LVAD) implantation.^9^ This poor correlation between LA and decompensated hemodynamics in advanced HF patients questions the prognostic ability of LA. With the broad spectrum of clinical presentations encompassing decompensated heart failure (DHF) and cardiogenic shock, a re‐examination of objective measurements of end‐organ perfusion and invasive hemodynamics is needed. We therefore sought to assess the relationship between LA levels, hemodynamics and clinical outcomes in patients with advanced heart failure presenting with clinical decline.

## 2 METHODS

This was a retrospective analysis of an invasive hemodynamics registry of advanced heart failure patients where providers were tasked to predict hemodynamics prior to right heart catheterization (RHC).^10^ Data for this current analysis were abstracted from this registry. The study took place at the University of Chicago Medical Center with patient data was collected between 2016 and 2018. Patients were eligible for inclusion if they had a history of heart failure with reduced ejection fraction, were >18 years of age, had a recorded admission LA level, and were presenting in either the outpatient or inpatient setting with apparent clinical decline or decompensation, as defined by worsening symptoms or objective markers consistent with worsening congestion or perfusion. Patients were excluded if they were mechanically ventilated or presented following cardiac arrest. Patients presenting with an acute coronary syndrome were excluded. Acute HF was defined as new onset (<1 month) HF with left ventricular (LV) cavity end‐diastolic dimensions^11^ <5.3 cm for women and <5.9 cm for men.^11^

**FIGURE 1:**
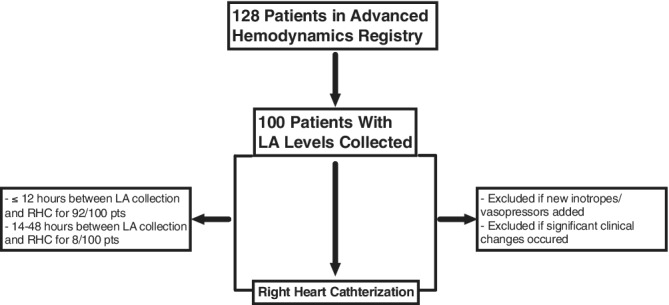
Study enrollment schema. ED, emergency department; HF, heart failure; LA, lactic acid; RHC, right heart catheterization

Outpatients were screened for study inclusion if they were evaluated in the clinic setting and sent directly for RHC if deemed necessary by the treatment team, specifically for precision‐guided management of HF. Pre‐RHC laboratory measurements were collected in the pre‐procedure holding area for patients admitted from the clinic. Inpatients were eligible for study inclusion if they presented to the emergency room or were transferred from an outside institution to either the inpatient floor or cardiac care unit for management. Patients with indwelling MCS devices were all admitted via transfer from outside institutions and had LA levels drawn after MCS placement and prior to RHC were eligible for study inclusion. The treatment team alone decided if RHC was necessary to guide management. Patients on existing inotropes, vasoactive agents or temporary MCS were also included. All laboratory assessments were collected on admission (first set collected during hospitalization) prior to RHC. Patients were excluded if significant clinical changes occurred between the time of LA measurement and RHC, including clinical deterioration or improvement defined as significant alterations in pre‐procedure medical therapy including addition or weaning of inotropes/vasopressors. Timing of LA collection and measurement of hemodynamics was carefully adjudicated and reported in the results (Figure 1). This study was approved by the University of Chicago Medical Center Institutional Review Board. All patients provided informed consent for registry inclusion.

INTERMACS (Interagency Registry for Mechanically Assisted Circulatory Support) classifications were made following RHC by the study investigators. Patients with liver disease were classified as having cirrhosis as documented in the presenting medical history; acute liver injury was defined as an elevation of transaminases three‐times the upper limit of normal plus elevation of total bilirubin without signs of encephalopathy.

LA was measured as a peripheral venous blood sample collected on ice at the University of Chicago clinical laboratory using the reagent from Roche laboratories. Verification of protocolized LA level collection and handling was completed by the study investigators through verbal confirmation with the central laboratory. The established normal reference range of LA is 0.9–2.1 mmol; we therefore defined LA levels >2.1 mmol/L as elevated.

Enrolled patients underwent supine RHC using standard technique. Hemodynamics collected included RA pressure, right ventricular (RV) pressure, pulmonary artery (PA) pressure, pulmonary capillary wedge pressure (PCWP), estimated Fick cardiac index, systemic and pulmonary vascular resistances. PCWP was recorded as the mean of a‐wave. Thermodilution cardiac output and index measurements were collected at the discretion of the primary operator performing the procedure. All measurements were obtained at end‐expiration. Estimated oxygen uptake was calculated through the Dehmer formula^12^: VO_2_ (ml/min) = 125 (ml/min/m^2^) × body surface area (BSA, m^2^). BSA was calculated according to the formula of Dubois.^13^ Estimated Fick cardiac index was then calculated by the following equation: [125 (ml/min/m^2^) × body surface area]/[(13.6*hemoglobin (g/L)* (ambient oxygen saturation – mixed venous saturation)]/BSA.

Statistics were performed using the Stata version 15 (StataCorp, Inc., College Station, TX). Descriptive statistics are reported as median (25%–75% interquartile range) when non‐normally distributed, and mean (standard deviation) when normally distributed. Comparison between groups of continuous variables were made using unpaired *t*‐test or Mann–Whitney *U*‐test as appropriate. Analysis of variance testing was used when comparing three continuous variables. Categorical data were compared between groups by Chi‐square analysis and Fischer's exact test when applicable. Assessment of correlation between hemodynamic variables and combines indices and LA was made by Spearman rank correlation. Proportion of patients with normal LA and abnormal hemodynamics was described for PCWP (>24 mmHg), CI (<2.2 L/min/m^2^), and pulmonary artery saturation (≤55%). These cut points were chosen given the high prevalence of primarily Stage D patients,^14^ with severely abnormal myocardial structure and function. Other measurements used were the ratio of systolic blood pressure (SBP) to PCWP, stratified above and below four^15^ and cardiac power index (CPI; {[mean arterial pressure (mmHg) × cardiac output (L/min/m^2^)] × [0.0022]}/[body surface area (m^2^)], stratified above and below the median.^16^ Pulmonary artery pulsatility index ([pulmonary artery systolic pressure ‐ pulmonary artery diastolic pressure]/[right atrial pressure]; PAPi) was calculated, stratified above and below the median, and correlated to LA levels.^17^, ^18^

A combined 30‐day composite endpoint of freedom from death or hospice discharge, inotrope dependence, heart transplantation or LVAD implantation was analyzed by Kaplan–Meier analyses and compared between normal and elevated LA groups using the log‐rank test. Outcomes at 30 days for patients discharged were adjudicated given uniform follow‐up clinical visits for all patients (non‐hospice) within the registry. Univariate Cox proportional hazard ratio analyses were performed to assess for significant predictors associated with 30‐day survival with medical therapy alone. Statistical significance was set at 0.05, and all tests were two‐tailed.

## 3 RESULTS

Patient characteristics for the overall cohort are shown in Table 1. Out of 128 patients within registry, 100 patients meet criteria for inclusion (Figure 1). Median patient age was 58 (47.3, 64.8) years, 70% were male, 39% were Caucasian. Median left ventricular ejection fraction was 22%^18^, ^28^; mean left ventricular end‐diastolic dimension was 6.4 (1.3) cm. Median LA prior to RHC was 1.6 (1, 2.1) mmol/L, with a range of 0.6–7.4 mmol/L (Appendix Figure 1). 57% of patients were on either inotropes and/or vasopressors prior to RHC. 7% (*N* = 7) of patients in total had percutaneous mechanical support devices (*N* = 6, Intra‐aortic balloon pump; *N* = 1, Impella) with inotropes and/or vasopressors prior to RHC. Median RA pressure was 15.5^(10,22)^; PCWP^(21,35)^ mmHg, PA oxygen saturation 54.5% (44.4, 61.5) and estimated Fick CI 2.0 (1.7, 2.5) L/min/m^2^. Eighty patients comprised the normal (≤2.1 mmol/L) LA group; 20 patients comprised the elevated LA (>2.1 mmol/L) group. Overall, the majority of patients in each subgroup had LA assays drawn within 4 h of RHC (Table 1). 13/20 (65%) in the elevated LA subgroup and 48/80 (60%) in the normal LA subgroup had LA levels drawn within 4 h of RHC. Eight patients had LA levels drawn greater than 12 h prior to RHC (range 14–48 h)—seven in the normal LA level group and one in the elevated LA group. The remaining LA levels were drawn within 12 h of RHC.

**TABLE 1:** Comparison of baseline characteristics between groups

Variable	Overall *(n* = 100)	LA >2.1 (*n* = 20)	LA ≤2.1 (*n* = 80)	*p‐*value
Age, median (IQR), y	58 (47.3, 64.8)	60 (49, 66.5)	57 (47, 64)	.76
Male, no (%)	70 (70)	13 (65)	57 (71.3)	.59
BMI, median (IQR), kg/m^2^	27.3 (23.6, 34.7)	23.7 (21.6, 28.9)	27.4 (24.5, 35.2)	.014^a^
Ischemic cardiomyopathy, no (%)	40 (40)	7 (35)	33 (41.3)	.80
Caucasian, no (%)	39 (39)	8 (45)	31 (38.8)	.92
LVEF, median (IQR), %	22 (18, 28)	22 (20, 24.8)	22 (17, 28.8)	.59
LVEDD, cm (*SD*)	6.4 (1.3)	6.3 (1.3)	6.4 (1.35)	.86
Chronic HF, no (%)	85 (85)	15 (75)	70 (87.5)	.16
Acute HF, no (%)	15 (15)	5 (25)	10 (12.5)	
Mode of admission, no. (%)				.10
Outside hospital transfer to ICU	35 (35)	7 (35)	28 (35)	
Outside hospital transfer to telemetry	19 (19)	0 (0)	19 (23.8)	
Emergency department presentation	33 (33)	12 (60)	41 (51.2)	
Clinic presentation	13 (13)	1 (5)	12 (15)	
INTERMACS classification, no (%)				.001^a^
INTERMACS 1	17 (17)	5 (25)	12 (15)	
INTERMACS 2	32 (32)	12 (60)	20 (25)	
INTERMACS 3+	51 (51)	3 (15)	48 (60)	
Implantable defibrillator, no (%)	65 (65)	12 (60)	53 (66.3)	.60
Sodium, median (IQR), mEq/L	137 (133, 139)	138 (134.3, 140)	136.5 (132.3139)	.35
Renal function on admission				
Creatinine, mg/dl	1.5 (1.1, 2.1)	1.5 (1.2, 2.1)	1.5 (1, 2.2)	.64
GFR, ml/min/1.73 m^2^	46 (31.3, 62.3)	46 (28.5, 56)	46.5 (31.3, 65.8)	.39
Serum LA, median (IQR), mmol/L	1.6 (1, 2.1)	3 (2.6, 3.7)	1.4 (1, 1.7)	<.001^a^
Timing of LA collection prior to RHC, no. (%)				.02^a^
<2 h	13 (13)	6 (30)	7 (8.8)	
<4 h	48 (48)	7 (35)	41 (51.3)	
<8 h	11 (11)	3 (15)	8 (10)	
<12 h	13 (13)	3 (15)	10 (12.5)	
>12 h	8 (8)	1 (5)	7 (8.8)	
NT‐proBNP, median (IQR), pg/mL	5871 (2747, 14 382)	11 911 (3900, 31 694)	5300 (2571, 13 067)	.11
Total bilirubin, median (IQR), mg/dL^b^	1.1 (0.7, 1.9)	1 (0.9, 2.4)	1.1 (0.7, 1.8)	.59
AST, median (IQR), U/L	33.5 (20.1, 61.5)	38 (17, 73)	33 (21, 61)	.78
Liver disease, no (%)				.02^a^
History of liver cirrhosis	5 (5)	1 (5)	4 (5)	
Acute liver injury	20 (20)	3 (15)	17 (21.3)	
Pre‐RHC therapy, no. (%)				.21
Oral medical therapy	36 (36)	5 (25)	31 (38.8)	
Inotropes and/or vasopressors	57 (57)	15 (75)	42 (52.5)	
IABP + inotropes/vasopressor	6 (6)	0 (0)	6 (6)	
Impella + inotrope/vasopressor	1 (1)	0 (0)	1 (1)	
30‐Day outcomes, no (%)				.58
Medical therapy alone	26 (26)	4 (20)	22 (27.4)	
LVAD	22 (22)	5 (25)	17 (23.8)	
Inotrope therapy	23 (23)	3 (15)	20 (20)	
Death	13 (13)	4 (20)	9 (12.5)	
Hospice	12 (12)	3 (15)	9 (11.3)	
Transplant	4 (4)	1 (5)	3 (5)	
SBP, median (IQR), mmHg	101 (92, 116)	93.5 (75.3, 118)	102 (93, 116)	.11
DBP, median (IQR), mmHg	68 (61, 75)	68 (60, 82)	68 (61, 74)	.78
MAP, median (IQR), mmHg	81.5 (71, 90.3)	76.5 (62.5, 91.5)	82 (71.3, 90.3)	.30
HR, median (IQR), bpm	92 (78, 105)	104.5 (81.8, 118.9)	90 (76.5, 103)	.036^a^
RAP, median (IQR), mmHg	15.5 (10, 22)	22.5 (14.3, 25)	15 (10, 20)	.013^a^
PCWP, median (IQR) mmHg	28 (21, 35)	30 (24.3, 38.3)	26 (21, 35)	.22
PA saturation, median (IQR), %	54.5 (44.4, 61.5)	42.1 (34.1, 53.6)	55.9 (48.5, 77.6)	<.001^a^
Ambient saturation, median (IQR), %	96 (96, 99)	96 (96, 98.5)	96 (96, 99)	.78
PASP, median (IQR), mmHg	56.5 (45.3, 69.8)	55 (41.3, 63)	58.5 (46.3, 70)	.28
PADP, median (IQR), mmHg	30 (25, 37)	35 (28.3, 38.9)	30 (23.5, 35)	.17
mPAP, median (IQR), mmHg	40 (32, 48)	44 (34, 47.3)	40 (31.3, 48.8)	.73
SVR, median (IQR), dyne⋅sec⋅cm^−5^	1240 (1004, 1618)	1463 (1036, 2029)	1232 (1001, 1511)	.17
Estimated Fick CI, median (IQR), L/min/m^2^	2 (1.7, 2.5)	1.7 (1.4, 2)	2 (1.7, 2.6)	.01^a^
SBP/PCWP ratio, median (IQR)	3.7 (2.9, 5.2)	3.56 (2.4, 4.3)	3.8 (3, 5.4)	.17
PAPi, median (IQR)	1.7 (1.2, 2.7)	1.8 (1.3, 2.9)	1.1 (0.7, 1.5)	.001^a^
CPI, median (IQR), W/m^2^	0.34 (0.28, 0.43)	0.28 (0.25, 0.31)	0.37 (0.31, 0.44)	.001^a^
Proportional pulse pressure, median (IQR)	33.8 (27.2, 40.7)	31.5 (22.2, 39.8)	35.8 (27.3, 66)	.2

Abbreviations: AST, asparate aminotransferase; BMI, body mass index; cm, centimeter; bpm, beats per minute; DBP, diastolic blood pressure; CI, cardiac index; CPI, cardiac power index; cm, centimeter; HF, heart failure; HR, heart rate; IABP, intra‐aortic balloon pump; INTERMACS, Interagency Registry for Mechanically Assisted Circulatory Support; ICU, intensive care unit; IQR, interquartile range; kg/m^2^, kilogram per meters squared; LA, lactic acid; LVEF, left‐ventricular ejection fraction; LVEDD, left‐ventricular end‐diastolic dimension; LVAD, left‐ventricular assist device; MAP, mean arterial pressure; mEq/L, milliequivalents per liter; mmol/L, millimoles per liter; mg/dl, milligrams per deciliter; ml/min/m^2^, milliliters per minute per meters squared; mmHg, millimeters of mercury; mPAP, mean pulmonary artery pressure; No, number; NT‐proBNP, N‐terminal pro brain natriuretic peptide; PA, pulmonary artery; PADP, pulmonary artery diastolic pressure; PAPi, pulmonary artery pulsatility index; PASP, pulmonary artery systolic pressure; PCWP, pulmonary capillary wedge pressure; RAP, right atrial pressure RHC, right heart catheterization; *SD*, standard deviation; SBP, systolic blood pressure; sec, second; SVR, systemic vascular resistance; y, year.

^a^ *p* < .05 by unpaired *t* test, Mann–Whitney *U* test or Chi‐square test/Fischer's exact test.

^b^ *n* = 93 patients with available admission NT‐proBNP.

Patients in the elevated LA level group had lower body mass index [27.3 (23.6, 34.7) kg/m^2^ vs. 27.4 (24.5, 35.2) kg/m^2^, *p* = .014], and proportionally a greater number of INTERMACS 1 and 2 patients (85% vs. 15%, *p* = .001). Acute liver injury was more common in the normal LA group (21.3% vs. 15%, *p* = .02). Other baseline demographic and laboratory variables were comparable between the two groups (*p* > .05 for all). In the elevated LA group, heart rate [104.5 (81.8, 118.9) bpm vs. 90 (76.5, 103) bpm, *p* = .036] was higher, while both pulmonary artery saturation [42.1% (34.1, 53.6) vs. 55.9% (48.5, 77.6), *p* < .001] and CPI [0.28 (0.25, 0.31) W/m^2^ vs. 0.37 (0.31, 0.44) W/m^2^, *p* < .001] were significantly lower than the normal LA groups (Table 1). PAPi was also significantly lower in the elevated LA group compared to the normal LA group [1.8 (1.3, 2.9) vs. 1.1 (0.7, 1.5), *p* = .001]. There were no other significant differences between hemodynamic variables observed between the two groups.

**FIGURE 2:**
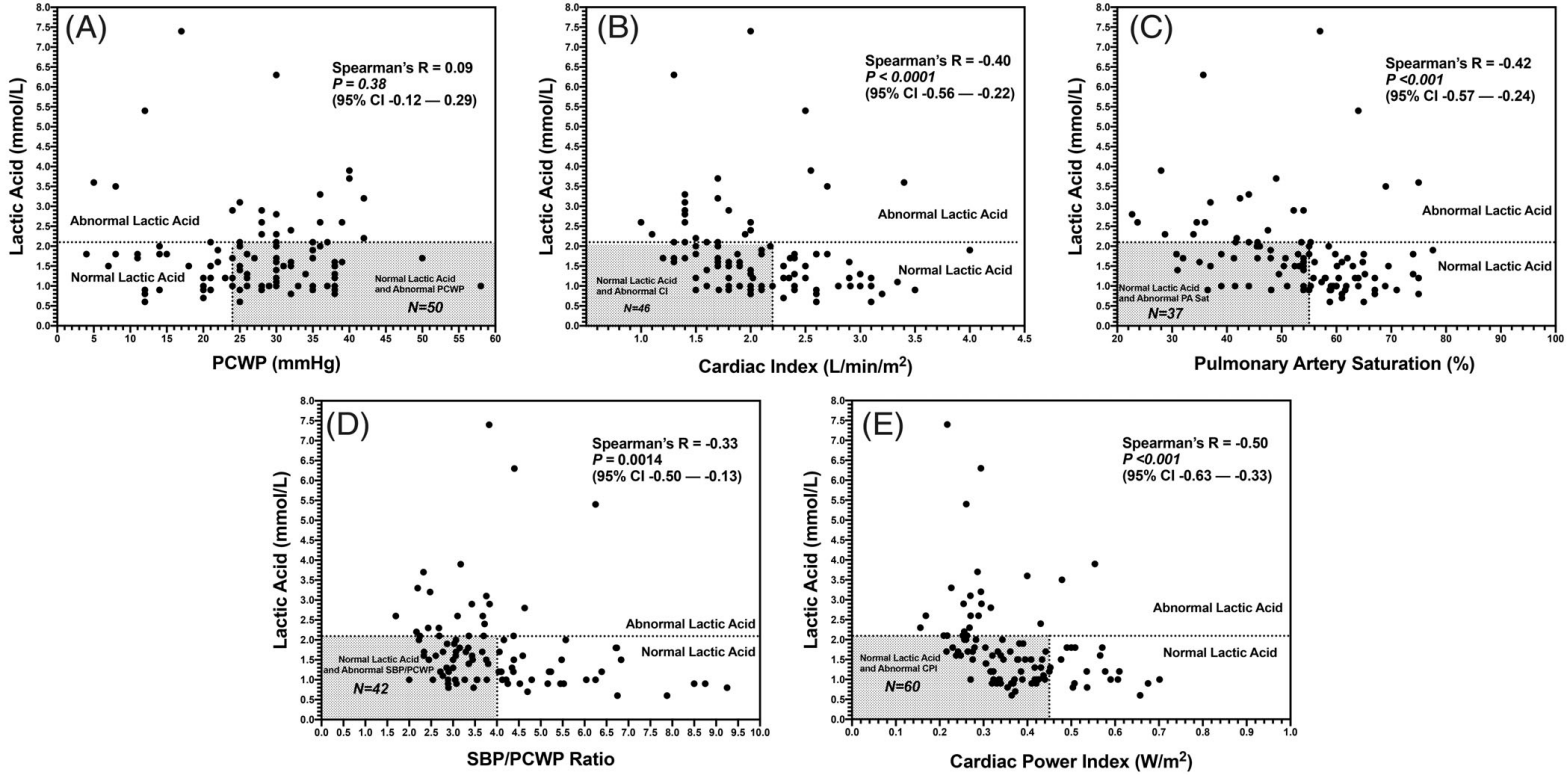
Correlation between LA levels and (A) PCWP (B) estimated Fick cardiac index (C) pulmonary artery saturation (D) SBP/PCWP ratio and (E) cardiac power index. Gray‐shaded areas indicate normal LA levels and abnormal hemodynamic values. LA, lactic acid; PCWP, pulmonary capillary wedge pressure; SBP, systolic blood pressure

Correlation plots between LA levels and hemodynamic variables are shown in Figure 2. For the overall cohort, there was no significant correlation between LA levels and PCWP (R = 0.09; 95% CI −0.12–−0.29, *p* = .38), with 50% of the overall cohort having PCWP >24 mmHg and LA levels ≤2.1. There was a significant, moderate inverse correlation between PA saturation and LA levels (R = −0.42; 95% CI −0.57–−0.24, *p* < .001), with 34% of the overall cohort having a PA saturation ≤55%. There was also a moderate, yet statistically significant inverse correlation between LA and estimated Fick CI (R = −0.40; 95% CI −0.56–−0.22, *p* < .001). However, 46% of the overall cohort had a LA level ≤2.1 and CI <2.2 L/min/m^2^. Three of four patients with LA level >2.1 and normal CI (≥2.2 L/min/m^2^) were vasoplegic with low filling pressures in the setting of infection despite a history of advanced HF and concern for DHF. The other patient had a low hemoglobin skewing the CI to the normal range in the setting of an elevated LA and elevated filling pressures with PCWP >24 mmHg. For patients with Thermodilution‐calculated CI (*N* = 72), there was a weak but statistically significant inverse correlation with LA (R = −0.32, 95% CI −0.52–−0.09, *p* = .006). 64% (*N* = 46) of this subgroup had a LA level ≤2.1 and a CI <2.2 L/min/m^2^ (Appendix Figure 2(A)). No significant correlation with systemic vascular resistance and LA level was observed (R = 0.16, 95% CI −0.04–0.35, *p =* .103; Appendix Figure 2(B)). There was a weak but statistically significant inverse correlation between LA and ratio of SBP/PCWP (R = ‐0.33; 95% CI −0.50–−0.13, *p* = .0014). However, 42% of the cohort had an SBP/PCWP ratio <4 and LA levels ≤2.1. There was also a moderate statistically significant inverse correlation between LA levels and CPI (R = −0.50; 95% CI −0.63–−0.33, *p* < .001). 29% of the overall cohort had LA levels ≤2.1 and CPI <0.34 W/m^2^. A weak but statistically significant inverse correlation between LA levels and proportional pulse pressure [(systolic pressure – diastolic pressure)/(systolic pressure)] was observed (R = −0.21; 95% CI −0.39–−0,006), *p =* .04; Appendix Figure 2(C)). 16% of the overall cohort had LA levels ≤2.1 and proportional pulse pressure <25%. A moderate inverse correlation between LA and PAPi (R = − 0.40; 95% CI −0.55–−0.22, *p* < .001; Appendix Figure 2(D)). 33% of the overall cohort had LA levels ≤2.1 and PAPi <1.7 (Abnormal PAPi <1.85).^17^

When the excluding the eight patients with RHC occurring >12 h post LA level collection, no new significant differences were observed in both primary correlation and outcome analyses (Appendix Figures 3 and 4).

**FIGURE 3:**
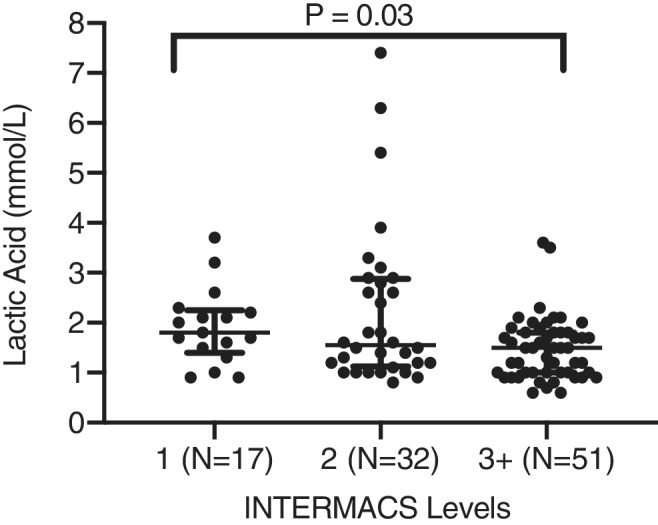
Relationship between LA levels and INTERMACS class. Centerline denotes median values with bars representative of interquartile range. INTERMACS, Interagency Registry for Mechanically Assisted Circulatory Support; LA, lactic acid

**FIGURE 4:**
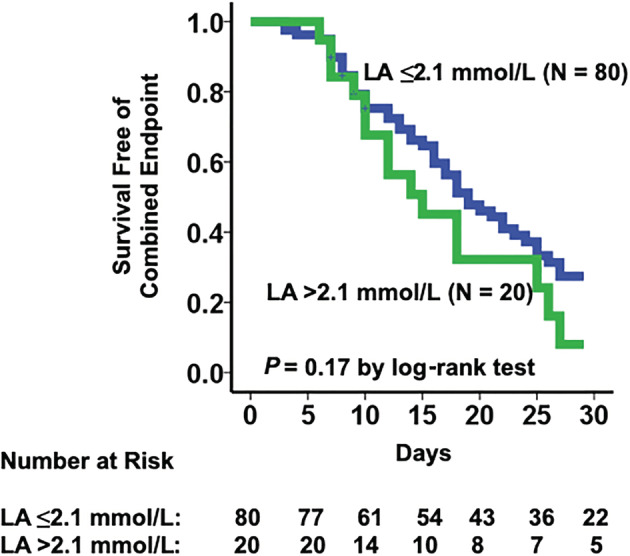
Comparison of 30‐day survival free of death/hospice, inotrope dependence, heart transplant and LVAD implantation between normal LA and abnormal LA groups. LA, lactic acid; LVAD, left‐ventricular assist device; mmol/L, millimoles per liter

Overall, LA levels were slightly greater in the INTERMACS 1 subgroup compared to groups 2 and 3 (Figure 3; *p* = .03). 40% (*N* = 32) of patients with normal admission LA levels (*N* = 80) were also classified as INTERMACS 1 or 2. All‐cause 30‐day mortality was 13%. 11% of the patients were discharged with hospice care, 26% survived on medical therapy alone, 24% remained inotrope dependent, 22% underwent LVAD implantation, and 4% underwent heart transplantation. In the additional 28 patients excluded from analysis due to lack of pre‐RHC LA assessment, 30‐day outcomes were as follows: medical therapy (*n* = 10, 35.7%); LVAD (*n* = 6, 21.4%); inotrope (*n* = 7, 25%); death (*n* = 4, 14.3%); hospice (*n* = 1, 3.6%). 30‐Day unadjusted survival free of death/hospice, inotrope dependence, progression to heart transplant/left‐ventricular assist device implant was 27.4% in the normal LA level group compared to 20% in the elevated LA group (*p* = .17, Figure 4). In the univariate Cox proportional hazard ratio analysis, only total bilirubin (per 1 mg/dL) was significantly associated with the combined clinical 30‐day endpoint (HR of 1.17, 95% CI 1.05–1.30; *p* = .005, Table 2). Dichotomized variables including PCWP ≥28 mmHg (HR of 1.99, 95% CI 0.81–5.1; *p* = .14) and PAPi <1.7 (HR 2.1, 95% CI 0.85–5.4; *p* = .11) by cut points determined by the sample median trended toward an association with the 30‐day endpoint (Table 2).

**TABLE 2:** Cox proportional hazard ratio analyses for 30‐day risk of death, hospice, inotrope dependence, heart transplantation or LVAD implantation

	Univariate hazard ratio (95% CI)	*p*‐Value
Demographics		
Age, yr	1.01 (0.99–1.02)	.63
Female	1.07 (0.65–1.76)	.78
BMI, kg/m^2^	0.99 (0.97–1.01)	.39
Ischemic cardiomyopathy	0.85 (0.53–1.36)	.50
Acute HF	0.68 (0.31–1.49)	.34
LEVDD, cm	1.12 (0.93–1.36)	.24
Hemodynamics		
Heart rate, bpm	1.01 (0.99–1.02)	.40
Systolic BP, mmHg	0.99 (0.99–1.01)	.40
RAP, mmHg	1.01 (0.98–1.04)	.46
PCWP, mmHg	1.01 (0.98–1.04)	.51
SBP / PCWP	0.94 (0.85–1.05)	.26
CPI, W/m^2^	0.21 (0.03–1.50)	.2
Estimated Fick CI <2.2 L/min/m^2^	1.47 (0.91–2.38)	.11
PCWP ≥28 mmHg	1.99 (0.81–5.1)	.14
PAPi <1.7	2.1 (0.85–5.4)	.11
Laboratory		
Lactic acid, mmol/L	1.17 (0.95–1.44)	.13
Sodium, mEq/L	1.01 (0.97–1.04)	.86
Creatinine, mg/dL	1.01 (0.78–1.30)	.95
Total bilirubin, per 1 mg/dL	1.17 (1.05–1.30)	.005
NT‐proBNP, pg/mL	1.00 (0.99–1.01)	.98

Abbreviations: BMI, body mass index; cm, bpm, beats per minute; CI, cardiac index; CPI, cardiac power index; HF, heart failure; HR, heart rate; kg/m^2^, kilogram per meters squared; LA, lactic acid; LVAD, left‐ventricular assist device; LVEDD, left‐ventricular end‐diastolic dimension; mEq/L, milliequivalents per liter; mmol/L, millimoles per liter; mg/dl, milligrams per deciliter; ml/min/m^2^, milliliters per minute per meters squared; mmHg, millimeters of mercury; NT‐proBNP, N‐terminal pro brain natriuretic peptide; PCWP, pulmonary capillary wedge pressure; PAPi, pulmonary artery pulsatility index; RAP, right atrial pressure; RHC, right heart catheterization; SBP, systolic blood pressure; y, year.

## 4 DISCUSSION

In this study, we analyzed the correlation of LA levels with invasive hemodynamics and clinical outcomes in a predominantly advanced heart failure cohort presenting with concern for DHF. Our main findings are:^1^ Grossly abnormal hemodynamics occurred frequently in the setting of normal LA levels (≤2.1 mmol/L)^2^; Advanced HF patients with normal admission LA levels in the setting of DHF overall had low survival with medical therapy alone at 30 days; and^3^ elevated admission LA levels in advanced HF patients was strongly associated with low survival with medical therapy alone at 30 days.

LA is predominantly produced in skeletal muscles and under aerobic conditions, is cleared by the liver. Under aerobic conditions, pyruvate is the end produce of glycolysis, while under anaerobic conditions, LA is an end‐product of glycolysis and a substrate for gluconeogenesis.^19^ During maximal exercise where there is a shift from aerobic to anaerobic metabolism, LA production rises significantly. Macrocirculatory and microcirculatory dysfunction leading to tissue hypoxia results in excess LA production, and decreased clearance due liver dysfunction from systemic hypoperfusion.^4^, ^20^ Several studies have demonstrated the association of elevated LA levels or poor clearance of LA with mortality in both ICU populations and those presenting with sepsis.^1^, ^21^, ^22^ However, only limited data have been published on the association of LA levels and cardiogenic shock or DHF, both of which are associated with high short‐term mortality.^18^, ^23^, ^24^, ^25^ Cardiogenic shock is conventionally defined by a systolic blood pressure less than 90 mmHg for greater than 30 min with elevated left ventricular filling pressures and clinical and serologic signs end‐organ hypoperfusion, including elevations in serum LA.^26^, ^27^, ^28^ The findings of clinical extremis are most likely to be present following acute myocardial infarction but may not be present in patients with acute on chronic heart failure presentations. Nonetheless, these patients may have grossly abnormal hemodynamics including a depressed CI and elevated PCWP while having normal serum LA. In chronic, advanced HF patients, physiologic adaptation to grossly elevated filling pressures may occur without any acute perturbations in end‐organ function. This was confirmed in a prior study of, nearly uniformly inotrope‐dependent end‐stage HF patients undergoing LVAD implantation who had RHC prior to surgery with corresponding LA levels measured; only 28% of the subgroup with CI ≤2.2 L/min/m^2^ had elevated LA levels^9^ and also in another study of patients with DHF where a modest inverse correlation between LA and Fick CI was observed but not with PCWP.^29^ Furthermore, the authors observed that elevated LA levels were associated with other markers of abnormal end‐organ perfusion and worsened INTERMACS classification, supporting the theory that acute on chronic HF presentations with elevated LA may be a late event. Our study is unique from prior evaluations of LA levels in acute decompensated heart failure (ADHF) given the heterogeneous nature of presentations with varying clinical trajectories, ~ 60% on inotropes and detailed correlative evaluation of LA with several hemodynamic variables and surrogate indices. Despite a depressed CI in these subgroups, oxygen delivery to peripheral tissues in resting states is likely maintained. However, these patients are unlikely to meet metabolic demands in moderate and above physical activity as reflected in their poor functional class.

We observed that normal LA levels are common and often discordant with abnormal hemodynamics in a cohort of predominantly chronic, advanced heart failure patients presenting with DHF. Nearly 50% of patients had normal LA levels in the setting of PCWP >24 mmHg and CI ≤2.2 L/min/m^2^. In the current study, we examined three surrogate hemodynamic indexes: Ratio of SBP to PCWP, CPI and proportional pulse pressure. SBP/PCWP<4 is associated with in‐hospital mortality and escalation to mechanical support in patients with acute coronary syndromes.^15^ CPI is associated with progression to heart transplant, LVAD and increased all‐cause mortality in advanced heart failure patients,^16^ while a proportional pulse pressure of <25% correlated with low CI.^30^, ^31^ In our cohort, more than 40% with normal LA had SBP/PCWP ratio <4, and 29% of patients with normal LA had a CPI below the cohort‐derived median of <0.34 W/m^2^. 20% of the normal LA subgroup had a proportional pulse pressure of <25%. Moreover, 40% of the normal LA group (*n* = 32) were classified as INTERMACS 1 or 2, which alone may confer an elevated risk of short‐term cardiovascular morbidity and mortality.^32^, ^33^

Normal LA in advanced heart failure patients presenting with DHF should not reassure the clinician that the patient is low‐risk in the short term. Under 30% of patients in with normal admission LA survived to 30 days on medical therapy alone, reflecting the high acuity of these patients despite having normal admission LA levels. LA was not a predictor of the composite heart failure clinical endpoint in this population. In the univariate analysis, only total bilirubin was modestly associated with increased risk of the composite 30‐day endpoint, but could not be confirmed in a multivariate analysis. Estimated Fick CI <2.2 L/min/m^2^, PCWP ≥28 mmHg and PAPi <1.7 trended toward an association in predicting risk of the composite 30‐day endpoint. The inherent nature of the study cohort comprised of advanced heart patients who were unable to tolerate medical therapy alone and vastly met the 30‐day endpoint (74%) biases the odds of individual variables to strongly affect observed risk. Traditionally, baseline PCWP and pre‐discharge filling pressures are strong predictors of longitudinal outcomes as shown in prior hemodynamic registries^18^, ^34^ Ultimately, a combination of multiple covariates together likely affects the aggregate clinical risk as no other single demographic, hemodynamic or laboratory variable was singularly risk‐modifying.

Compared to patients with normal admission LA, those with elevated LA on presentation had a high rate of 30‐day mortality (20%). This subgroup had significantly higher baseline heart rate, right atrial pressure, along with significantly lower pulmonary artery saturation, Fick‐estimated CI, and CPI. Furthermore, the calculated organ perfusion pressure, as determined by the difference between mean arterial pressure and right atrial pressure, was significantly lower in the elevated LA cohort. This generally corresponds with increased right sided congestion which may affect LA clearance. Lower mean BMI was observed in the elevated LA group, which may be attributed to non‐normal sample distribution and unbalanced sample size, as there is no obvious physiologic reason. Additionally, 70% of this subgroup was classified as INTERMACS 1 or 2. Therefore, the presence of elevated LA levels in patients with DHF should appropriately alert the clinician to acutely escalate therapy or expedite diagnostic testing.

Serum LA >3.0 mmol/L has been incorporated in a novel risk score along with other clinical and hemodynamic parameters for patients presenting with cardiogenic shock,^35^ which improved outcomes due to early recognition. In NYHA Class IV patients who presents with DHF, elevation of LA may represent a critical inflection point of markedly increased mortality risk unless intervention is initiated. Though validated as a marker of critical illness when elevated, elevated LA levels may not solely represent tissue hypoxia and instead also signal a physiologic stress response with increased sympathetic nervous system activation inducing a state of accelerated glycolysis and modified bioenergetic supply.^36^ Challenges remain, however, for early recognition of the patient with cardiogenic shock who presents with a normal LA. More liberal use of hemodynamic assessment may be needed to better classify NYHA Class III‐IV patients presenting with ADHF.^37^ Early RHC should be considered in these patients even when clinical assessment, and particularly the physical exam, is not otherwise indicative of cardiogenic shock.^10^

## 5 LIMITATIONS

This study has several limitations. There was a time gap between measurement of LA levels and hemodynamic assessment, although patients were excluded if significant therapeutic changes occurred during this time period. Eight patients had LA drawn >12 h (range 14–48 h). Admission LA levels in advanced HF patients presenting with ADHF were studied to assess the proximal relationship with initial invasive hemodynamic and risk of progression to short term clinical endpoints. The trend in LA level changes over the course of the hospitalization was not evaluated, though holds importance in understanding overall clinical acuity. A majority of patients presented on existing inotropic support and/or MCS prior to RHC precluding generalization of the findings to patients only receiving oral medical therapy on presentation.

This cohort was a highly‐selected, convenience sample comprised predominantly of advanced HF patients, and the results may not be applicable to other populations. Accurate comparison of baseline covariates and clinical outcomes between the normal and elevated LA groups is limited by an unbalanced sample size. Furthermore, subclassification of outcomes including progression to heart transplantation and LVAD implantation is center and provider dependent, and therefore may not be generalizable. The majority of the patients in the advanced heart failure cohort met the primary endpoint which clearly affected the ability for assessment of clinical risk predictors which would have had an association with the composite outcome of interest. This therefore undoubtedly affected the ability to perform multivariate modeling. We calculated an estimated Fick CI^12^ to establish the cut‐off of 2.2 L/min/m^2^ which can be flawed when compared to direct measurement,^38^ though may be more favorable compared to thermodilution‐derived CI due to the high prevalence of tricuspid regurgitation in this population.

## 6 CONCLUSIONS

In patients with advanced HF presenting with DHF, a normal LA on presentation was remarkably common and may indicate that the basic metabolic demands of the body are still being met despite severely reduced cardiac function. However, normal LA levels may not accurately discriminate the clinical severity of presentation in advanced HF patients as defined by invasive hemodynamic. Furthermore, patients of this type may be in overt cardiogenic shock with a normal LA, along with having a high short‐term mortality risk and need for advanced heart failure therapies. Elevated LA levels in these patients, however, carry more certainty of immediate high clinical acuity and should be triaged appropriately to better mitigate adverse outcomes.

## CONFLICT OF INTEREST

Nir Uriel receives grant support and consulting fees from Abbott Healthcare, Leviticus Cardio, and Medtronic. Daniel Burkhoff has received an unrestricted educational grant from Abiomed. Valluvan Jeevanandam receives consulting fees from Abbott Healthcare.

## Supporting information


**Appendix Figure 1** Distribution of LA levels in the overall cohort. Abbreviations: mmol/L, millimoles per liter.

**Appendix Figure 2** Correlation between LA levels and (A) Thermodilution Cardiac Index (*n* = 72 patients) (B) Fick Formulae‐Derived Systemic Vascular Resistance (C) Proportional Pulse Pressure and (D) Pulmonary Artery Pulsatility Index. Gray‐shaded areas indicate normal LA levels and abnormal hemodynamic values. Abbreviations: CI, cardiac index; cm, centimeter; sec, second.

**Appendix Figure 3** Correlation between LA levels and (A) PCWP (B) Estimated Fick Cardiac Index (C) Pulmonary Artery Saturation (D) SBP/PCWP Ratio and (E) Cardiac Power Index in patients with LA level collection and RHC within 12 h (*n* = 92 patients). Gray‐shaded areas indicate normal LA levels and abnormal hemodynamic values. Abbreviations: PCWP, pulmonary capillary wedge pressure; SBP, systolic blood pressure.

**Appendix Figure 4** Comparison of 30‐day survival free of death/hospice, inotrope dependence, heart transplant and LVAD implantation between normal LA and abnormal LA groups in patients with LA level collection and RHC within 12 h (*n* = 92 patients). Abbreviations: LA, lactic acid; mmol/L, millimoles per liter.
