# Supplementary figures and images for: Discordance between lactic acidemia and hemodynamics in patients with advanced heart failure
Source: Clin Cardiol. 2021 Mar 18;44(5):636–45. doi: 10.1002/clc.23584 (PMC8119805; doi:10.1002/clc.23584)

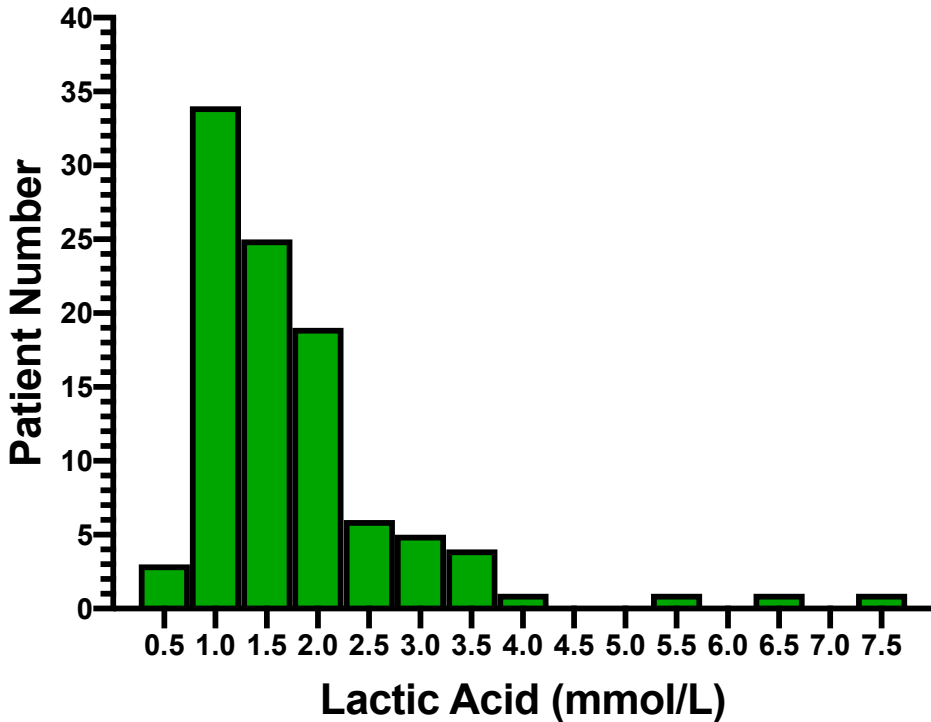

Supplement: Supplementary file 1 — Appendix Figure 1 Distribution of LA levels in the overall cohort. Abbreviations: mmol/L, millimoles per liter. [file CLC-44-636-s004.pdf]

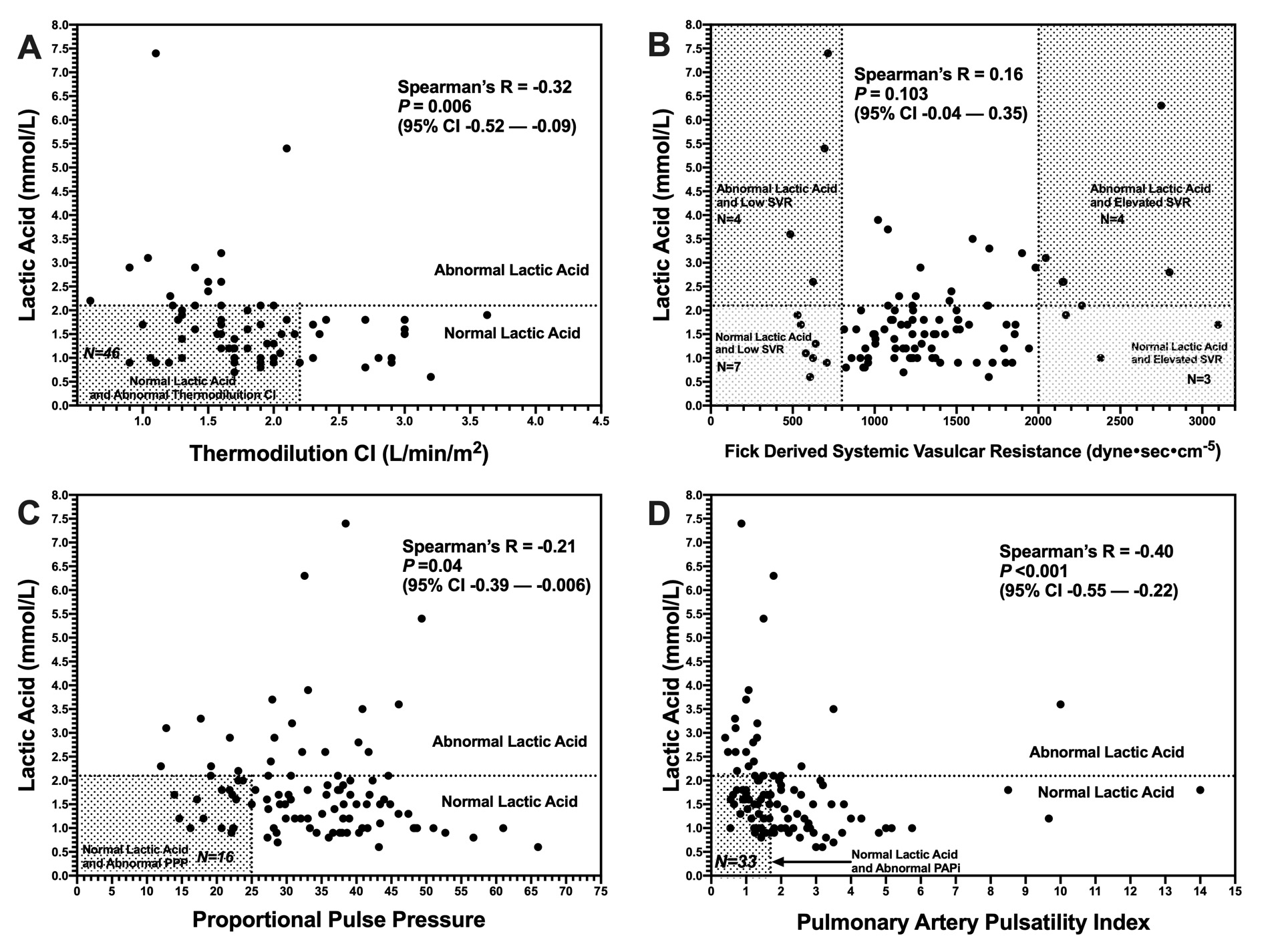

Supplement: Supplementary file 2 — Appendix Figure 2 Correlation between LA levels and (A) Thermodilution Cardiac Index (n = 72 patients) (B) Fick Formulae‐Derived Systemic Vascular Resistance (C) Proportional Pulse Pressure and (D) Pulmonary Artery Pulsatility Index. Gray‐shaded areas indicate normal LA levels and abnormal hemodynamic values. Abbreviations: CI, cardiac index; cm, centimeter; sec, second. [file CLC-44-636-s002.jpg]

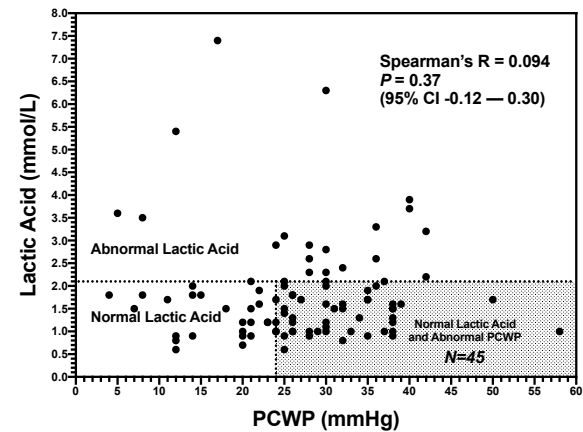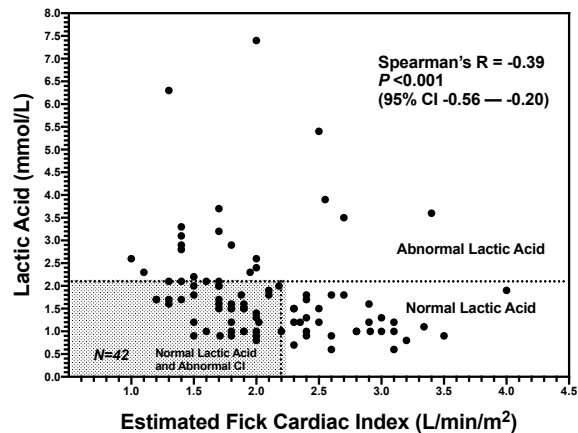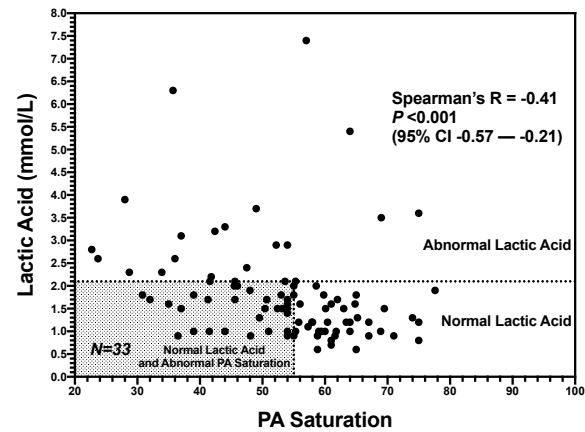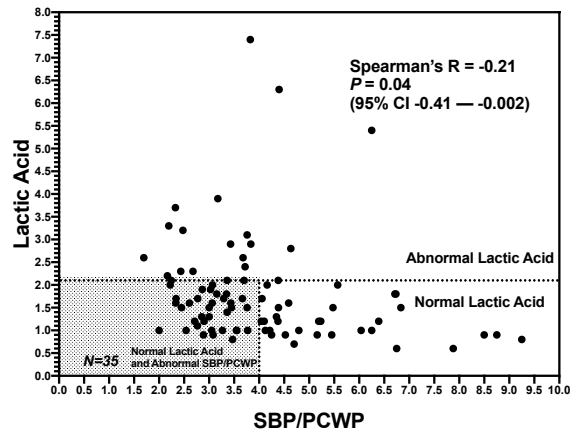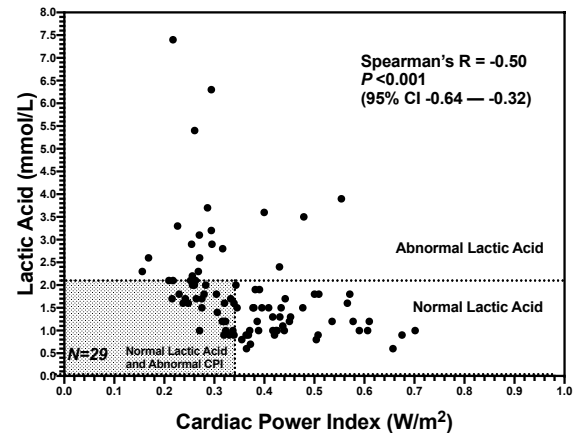

Supplement: Supplementary file 3 — Appendix Figure 3 Correlation between LA levels and (A) PCWP (B) Estimated Fick Cardiac Index (C) Pulmonary Artery Saturation (D) SBP/PCWP Ratio and (E) Cardiac Power Index in patients with LA level collection and RHC within 12 h (n = 92 patients). Gray‐shaded areas indicate normal LA levels and abnormal hemodynamic values. Abbreviations: PCWP, pulmonary capillary wedge pressure; SBP, systolic blood pressure. [file CLC-44-636-s003.pdf]

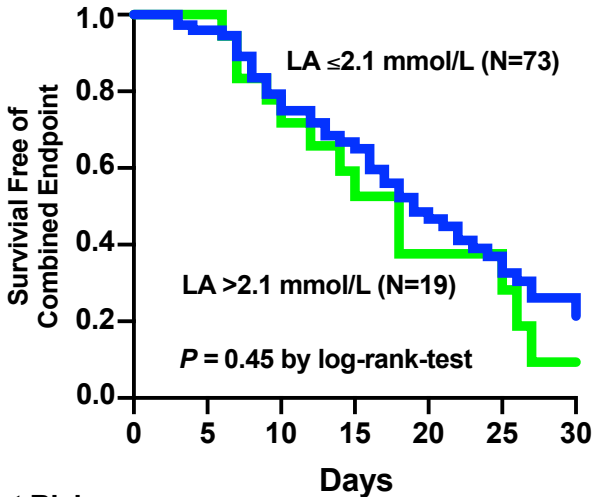

Number at Risk

|                      |    |    |    |    |    |    |    |
|----------------------|----|----|----|----|----|----|----|
| LA $\leq 2.1$ mmol/L | 73 | 70 | 56 | 50 | 40 | 33 | 19 |
| LA $> 2.1$ mmol/L    | 19 | 19 | 14 | 11 | 9  | 8  | 6  |

Supplement: Supplementary file 4 — Appendix Figure 4 Comparison of 30‐day survival free of death/hospice, inotrope dependence, heart transplant and LVAD implantation between normal LA and abnormal LA groups in patients with LA level collection and RHC within 12 h (n = 92 patients). Abbreviations: LA, lactic acid; mmol/L, millimoles per liter. [file CLC-44-636-s001.pdf]
